# Enhanced Identification of Novel Potential Variants for Appendicular Lean Mass by Leveraging Pleiotropy With Bone Mineral Density

**DOI:** 10.3389/fimmu.2021.643894

**Published:** 2021-04-06

**Authors:** Cheng Peng, Feng Liu, Kuan-Jui Su, Xu Lin, Yu-Qian Song, Jie Shen, Shi-Di Hu, Qiao-Cong Chen, Hui-Hui Yuan, Wen-Xi Li, Chun-Ping Zeng, Hong-Wen Deng, Hui-Ling Lou

**Affiliations:** ^1^ Department of Geriatrics, National Key Clinical Specialty, Guangzhou First People’s Hospital, School of Medicine, South China University of Technology, Guangzhou, China; ^2^ Center for Bioinformatics and Genomics, Department of Global Biostatistics and Data Science, Tulane University, New Orleans, LA, United States; ^3^ Shunde Hospital of Southern Medical University (The First People’s Hospital of Shunde), Foshan City, China; ^4^ Department of Endocrinology and Metabolism, The Third Affiliated Hospital of Southern Medical University, Guangzhou, China; ^5^ Department of Endocrinology and Metabolism, The Fifth Affiliated Hospital of Guangzhou Medical University, Guangzhou, China

**Keywords:** appendicular lean mass, bone mineral density, pleiotropy, genome wide association study, novel SNPs

## Abstract

Strong relationships have been found between appendicular lean mass (ALM) and bone mineral density (BMD). It may be due to a shared genetic basis, termed pleiotropy. By leveraging the pleiotropy with BMD, the aim of this study was to detect more potential genetic variants for ALM. Using the conditional false discovery rate (cFDR) methodology, a combined analysis of the summary statistics of two large independent genome wide association studies (GWAS) of ALM (n = 73,420) and BMD (n = 10,414) was conducted. Strong pleiotropic enrichment and 26 novel potential pleiotropic SNPs were found for ALM and BMD. We identified 156 SNPs for ALM (cFDR <0.05), of which 74 were replicates of previous GWASs and 82 were novel SNPs potentially-associated with ALM. Eleven genes annotated by 31 novel SNPs (13 pleiotropic and 18 ALM specific) were partially validated in a gene expression assay. Functional enrichment analysis indicated that genes corresponding to the novel potential SNPs were enriched in GO terms and/or KEGG pathways that played important roles in muscle development and/or BMD metabolism (adjP <0.05). In protein–protein interaction analysis, rich interactions were demonstrated among the proteins produced by the corresponding genes. In conclusion, the present study, as in other recent studies we have conducted, demonstrated superior efficiency and reliability of the cFDR methodology for enhanced detection of trait-associated genetic variants. Our findings shed novel insight into the genetic variability of ALM in addition to the shared genetic basis underlying ALM and BMD.

## Introduction

Sarcopenia, a “geriatric giant” characterized by progressive loss of muscle mass and strength, is associated with increased risk of falls, frailty and disability. Appendicular lean mass (ALM) is a major index that defines sarcopenia (1), a condition that causes heavy economic burden to healthcare systems, the annual cost of which in the United States alone is more than 18 billion dollars (2). Sarcopenia is a highly heritable complex disease, lean mass (LM), having a heritability estimated to be 0.52–0.60 (3, 4). Previous genome wide association studies (GWAS) have identified a number of loci and genes associated with LM. For example, loci are present in *TRHR* (5), *GLYAT* (6), *PRDM16* (7) and *TNRC6B* (8). Furthermore, the latest large-scale GWAS has just identified and replicated five loci strongly associated with LM at a genome-wide level of significance (9), and a recent ALM GWAS study identified 1059 conditionally independent variants from 799 loci with a genome-wide significance level (p <5 × 10^−9^) using the UKB cohort (10). These findings help us better understand the genetic background of sarcopenia, but because of the high heritability of LM, the current findings are extremely limited, a missing heritability is evident. To further elucidate the genetic mechanisms of sarcopenia, deeper exploration of LM-associated genetic variants are required.

Epidemiological and clinical studies have shown multiple relationships between LM and bone mineral density (BMD). Both are complex traits with high degrees of genetic determination. The estimated heritability of BMD is 0.75–0.83 (11, 12). Sarcopenia and osteoporosis are both diseases related to aging, the loss of muscle mass and strength decreases the mechanical load on bone, thus causing a reduction in BMD (13). It was reported that hip BMD is positively associated with skeletal muscle strength and mass (13, 14). BMD and LM share common risk factors, such as aging, long-term confinement in bed and inflammatory factors. The same molecular pathways were also reported to be associated with both osteoporosis and sarcopenia, for example, the Wnt signaling pathway (15, 16). The underlying mechanism of the association between LM and BMD remains unclear. We hypothesize that it might partially result from the pleiotropic effect, namely that a single gene can influence multiple traits. In 2017, the pleiotropic effect of LM and BMD were first reported at the locus *SREBF1/TOM1L2* in the pediatric population (17). Although the findings were limited, it strongly supported our hypothesis of pleiotropy between LM and BMD. By leveraging the power of pleiotropy and using appropriate analytical methods we may ascertain a greater number of potential pleiotropic variants in addition to trait-associated variants for ALM and/or BMD.

Recently, an analytical method—pleiotropy-informed conditional false discovery rate (cFDR)—was successfully applied to GWAS datasets of correlated phenotypes. According to the simulation, while comparing to the unconditional FDR method, cFDR method was able to increase the discovery of non-null trait-associated single nucleotide polymorphisms (SNP) by 15–20 fold during single phenotype analysis (18). The cFDR methodology provides the opportunity to identify a greater number of novel potential variants in addition to pleiotropic variants for highly correlated phenotypes without recruiting additional samples (18, 19). In our recent studies, we successfully implemented the cFDR methodology on a number of correlated phenotypes. Not only did we discover novel potential genetic variants for single phenotypes, we also uncovered potential common variants for highly-associated traits, such as BMD with height, birth weight with type 2 diabetes, and BMD with coronary heart disease (19–21). In this study, we utilized the cFDR methodology in two independent large scale GWAS datasets of ALM and BMD, aiming to find additional novel potential genetic variants for ALM in addition to potential common variants for both ALM and BMD.

## Methods and Materials

### ALM and BMD GWAS Datasets

ALM and BMD GWAS summary statistics were obtained from the Genetic Factors for Osteoporosis Consortium (GEFOS). Both sets of data were released in 2017. The ALM datasets contain summary statistics from adolescent populations of European, African American and Korean descent (n = 73,420), including 2,699,262 SNPs (9). The BMD datasets contain summary statistics of total body BMD (excluding the head) from European and African American populations in four pediatric cohort studies (n = 10,414), including a total of 2,276,810 SNPs (17). Genetic factors are the principal determinants of BMD at any age, with BMD in childhood highly correlated with peak BMD in adulthood (22). ALM and BMD GWASs are independent with no overlapping subjects. The inclusion criteria and phenotypic characteristics of ALM and BMD GWASs are described in detail in the original publications (9, 17)

### Data Processing

Based on HapMap 3 genotypes and the linkage disequilibrium (LD) SNP pruning method, we pruned ALM and BMD data using PLINK software. The linkage disequilibrium (LD) value for each pair of SNPs was calculated. For SNP pairs with r^2^ >0.2, the SNP with the smaller minor allele frequency (MAF) was deleted so that no pairs of SNPs with high LD remained (18). After pruning, there were 1,098,760 SNPs remaining for ALM and 996,515 SNPs for BMD. The ALM data was then combined with the BMD data based on common SNPs, resulting in 976,149 SNPs remaining for subsequent cFDR analysis. The direction of effect of an SNP on ALM was ascertained from the “Effect” entry in the original ALM data, and ascertained by the “Beta” value for BMD in the original BMD data. If the Beta value of the SNP was higher than 0.00, the direction of effect of the SNP was termed “+”, if the Beta value was lower than 0.00, the direction was “−”. “+” indicates a positive effect of the reference allele, “−” indicating a negative effect of the reference allele. Genomic control was conducted in the original GWASs so that the estimated variance of each SNP would not be inflated due to the structure of the population (9, 17).

### Statistical Analysis

#### Estimation of Pleiotropic Enrichment

To evaluate whether the primary phenotype was related to the phenotype under study by the null hypothesis, a quantile–quantile plot (Q–Q plot) was created using the “ggplot2” module in R software. Q–Q plot is a graphical tool that represents pleiotropic enrichment between the nominal and empirical traits. Nominal −log_10_(p) values are plotted on the Y-axis and the empirical quantiles (−log_10_(q)) on the X-axis. The Q–Q curve is plotted for the quantile of nominal −log_10_(p) values for association of the subset of variants that are below differential significance thresholds in the conditional trait. Pleiotropic enrichment can then be observed intuitively as each curve gradually deviates up leftwards from the expected identify line.

#### Calculation of cFDR and Conjunction cFDR (ccFDR)

The cFDR methodology was developed from that of traditional FDR (23). Its value relates to the false probability that a random SNP is not associated with the principal phenotype given that the p-value of this SNP for the principal phenotype and conditional phenotype are both less than two pre-defined trait-specific significance thresholds (18). In this study, we computed a cFDR value for each SNP while referencing ALM as the principal phenotype and BMD as the conditional phenotype (ALM|BMD), then in reverse, with BMD conditional on ALM (BMD|ALM). The ccFDR was computed as the maximum cFDR value of the two phenotypes, referring to the false possibility that the SNP is associated with neither of the two phenotypes, and used to identify potential pleiotropic SNPs that are associated with both phenotypes. cFDR and ccFDR thresholds were set to 0.05. SNPs with cFDR or ccFDR values lower than 0.05 were considered significantly associated with the corresponding phenotype or both phenotypes, with the false discovery rate lower than 0.05. The detailed procedure for calculating cFDR and ccFDR is described in previous publications (19, 20). To mark the chromosomal positions and significance of cFDR or ccFDR values of the SNPs, we used R software to plot cFDR and ccFDR Manhattan plots. Significant cFDR or ccFDR SNPs were plotted above the red line at 1.3 (corresponding to cFDR or ccFDR values lower than 0.05).

#### Annotation of Novel Potential Pleiotropic SNPs and Potential ALM-Associated SNPs

To verify whether the SNPs significant in cFDR and ccFDR were novel, we compared them with previous GWAS findings. SNPs that had been confirmed to be associated with only ALM and BMD were not available, so we downloaded the SNPs that had been confirmed to be associated with LM and BMD in previous GWASs (**Supplementary Table 2**), and SNPs associated with LM (**Supplementary Table 4**) from the European Bioinformatics Institute website (https://www.ebi.ac.uk/gwas, Accessed Jan 2020). Based on HapMap3 and using *R* ^2^ 0.8 as a threshold (24), we then inputted SNPs significant in ccFDR or cFDR and the SNPs previously confirmed by GWAS into the SNP Annotation and Proxy Search tool (SNAP, http://archive.broadinstitute.org/mpg/snap) and Ldlink (https://ldlink.nci.nih.gov/?tab=home) to perform LD analysis. Only SNPs not clustered in the same LD block with previous GWAS findings were considered as potential novel SNPs, otherwise they were regarded as replication of previous GWAS findings. LD block refers to genomic segment consisting of adjacent genetic polymorphisms with high probability (e.g. *R* ^2^ >0.8) of non-random association of alleles.

#### Gene Expression Validation Analysis

Gene expression profiling of mRNA levels in vastus lateralis muscle biopsies was used to explore whether genes corresponding to potential ALM-associated SNPs were expressed differentially between young and old adults (25). A Wilcoxon Rank Sum test, which is a rank-based non-parametric test that compares two groups of expression profiles with no assumption of normality, was computed for the mRNA levels of the genes in vastus lateralis muscle biopsies from 16 young adults (eight men and eight women, mean age: 24 years) and 12 old adults (six men and six women, mean age: 84 years). Details of the gene expression profiling of the muscle biopsies and how it was conducted are described in the original publication (25).

In addition to the vastus lateralis muscle biopsies, gene expression validation analysis was also performed on bone tissue for genes corresponding to potential pleiotropic SNPs that had been identified by ccFDR. Using gene expression profiling in iliac bone biopsies from 84 postmenopausal Caucasian women (26), the correlation coefficients were calculated of whole body BMD against mRNA levels of genes significant by ccFDR. Details of the gene expression profiling of iliac bone biopsies is described in the original publication (26).

#### Protein–Protein Interaction Analysis and Functional Term Enrichment Analysis

SNPs identified by cFDR were mapped to their corresponding genes using the online tool SNPinfo (https://snpinfo.niehs.nih.gov/). The ALM-associated genes found to be significant by cFDR were inputted into the online database STRING 10.0 (http://string-db.org) to perform protein-protein interaction (PPI) analysis, enabling further exploration of the biological functional role of the proteins produced by ALM-associated genes. Functional term enrichment analysis was conducted using the Web-Based Gene Set Analysis Toolkit (27), assisting in partial validation of results by discovering gene sets in gene ontology (GO) terms and KEGG canonical pathways related to muscle development and/or bone metabolism. GO categories were pre-filtered for redundancy using the online tool REVIGO (28). For GO and KEGG enrichment analysis, an adjusted p value (adjP) lower than 0.05 was regarded as significant. adjP is the p value adjusted by multiple test adjustments.

## Results

### Pleiotropic Enrichment Assessment and Novel Potentially Pleiotropic SNPs Identified by ccFDR

**Figure 1:**
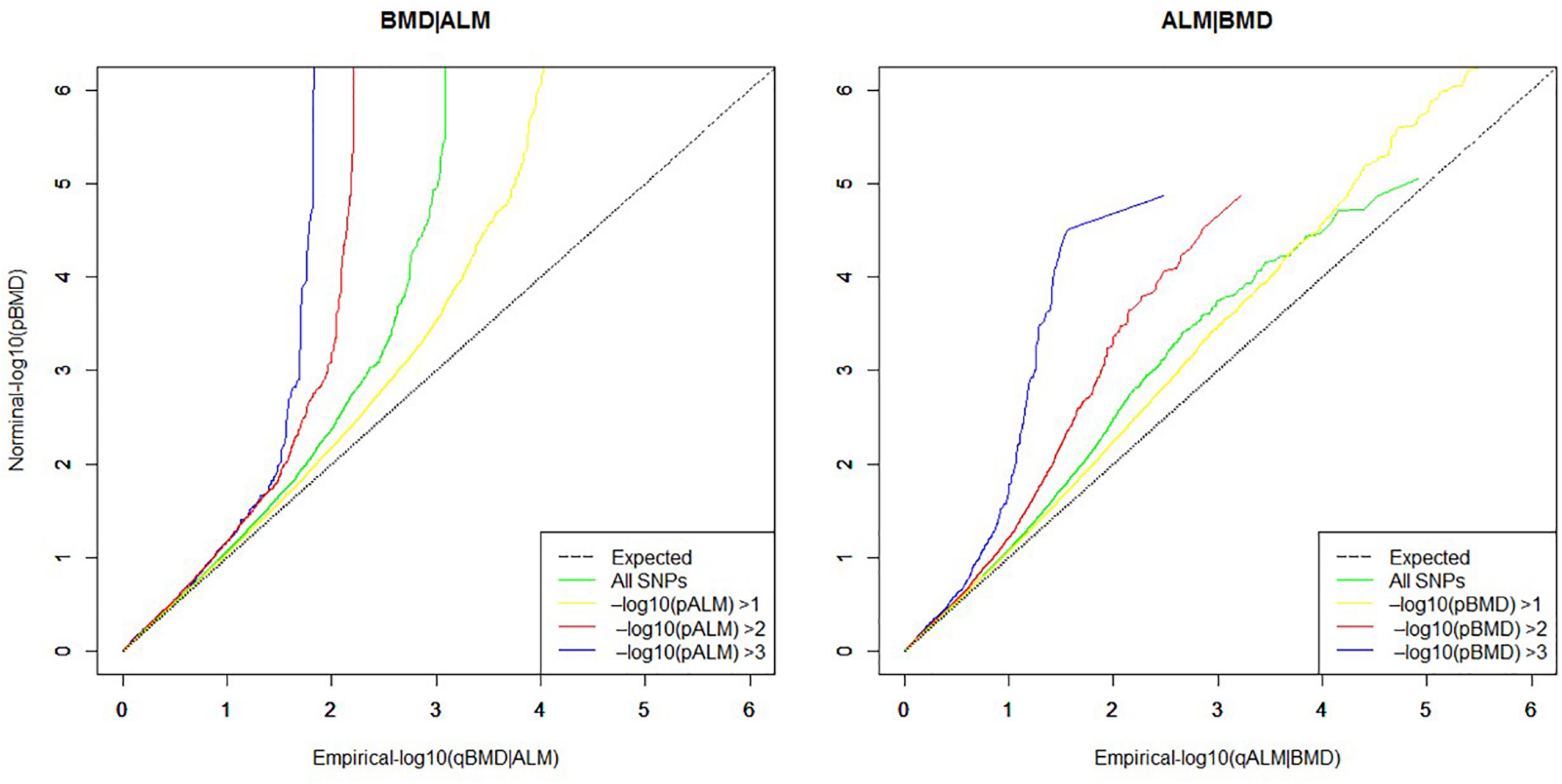
Quantile–quantile plots (Q–Q plots) for ALM as a function of significance of association with BMD (ALM|BMD), and as the reverse, BMD as a function of significance of association with ALM (BMD|ALM).

As presented in **Figure 1**, the curves gradually diverge from the expected baseline, with strong pleiotropic enrichment observed for ALM SNPs across different levels of association with BMD (ALM|BMD) in addition to the converse, for BMD SNPs conditioning on ALM (BMD|ALM). A total of 55 potential pleiotropic SNPs mapping to five chromosomes were identified by ccFDR that associated with both ALM and BMD (**Supplementary Figure 1** and **Supplementary Table 1**). We compared the results from this study with the 12 pleiotropic SNPs reported by a recent bivariate GWAS meta-analysis of pleiotropic effect on LM and BMD (**Supplementary Table 2**). Interestingly, two previously reported pleiotropic SNPs, rs12284933 and rs754388, were replicated in the present study (SNPs marked * in **Supplementary Table 1**). Twenty seven potentially pleiotropic SNPs identified by ccFDR resided in the same LD block as the previously reported 12 pleiotropic SNPs (SNPs marked ** in **Supplementary Table 1**). These 29 SNPs identified by ccFDR are considered a successful replication of previous GWAS findings. A total of 26 novel potential pleiotropic SNPs mapping to five chromosomes were identified by ccFDR for ALM and BMD (**Table 1**). Some 16 of these 26 novel potential pleiotropic SNPs have the same direction of effect on both ALM and BMD. Of the 26 novel potential pleiotropic SNPs, there are eight SNPs located in *LRP5*, seven in *PPP6R3*, four near *RUNX2* and *CLIC5*, and three SNPs near *BMP2* and *FUSIP1P2.* Interestingly, the loci *PPP6R3* and *RIN3* have been previously reported in a previous GWAS analysis to be associated with both BMD and LM (17).

**Table 1:** Novel potential pleiotropic SNPs for ALM and BMD identified by ccFDR.

SNP	Chr	Position	ccFDR	Mapped Gene	Effect		Gene Expression validation	
					ALM	BMD	p.ALM	p.BMD
rs1159531	20	7051595	3.53E−02	BMP2/FUSIP1P2	+	+	1.48E−01/None	7.96E−02/None
rs1159530	20	7051709	4.41E−02	BMP2/FUSIP1P2	+	+	1.48E−01/None	7.96E−02/None
rs1884303	20	7054353	4.72E−02	BMP2/FUSIP1P2	−	+	1.48E−01/None	7.96E−02/None
rs9472536	6	45724421	1.08E−02	RUNX2/CLIC5	−	+	1.34E−01/**7.55E**−**06**	**1.31E**−**02/2.39E**−**03**
rs6923368	6	45727343	8.66E−03	RUNX2/CLIC5	−	+	1.34E−01/**7.55E**−**06**	**1.31E**−**02/2.39E**−**03**
rs16873645	6	45773425	1.22E−02	RUNX2/CLIC5	+	+	1.34E−01/**7.55E**−**06**	**1.31E**−**02/2.39E**−**03**
rs12333018	6	45782629	2.16E−02	RUNX2/CLIC5	+	+	1.34E−01/**7.55E**−**06**	**1.31E**−**02/2.39E**−**03**
rs648732	11	65387743	4.83E−02	MUS81	−	+	9.53E−01	**2.88E**−**02**
rs3758938	11	67158938	3.50E−02	TBX10	−	−	4.14E−01	7.68E−01
rs2306862	11	67934086	3.19E−03	LRP5	−	+	2.34E−01	1.66E−01
rs314750	11	67938604	9.83E−03	LRP5	+	+	2.34E−01	1.66E−01
rs923346	11	67938951	3.60E−03	LRP5	+	+	2.34E−01	1.66E−01
rs599083	11	67948922	1.46E−02	LRP5	+	+	2.34E−01	1.66E−01
rs556442	11	67949266	3.26E−02	LRP5	+	+	2.34E−01	**1.66E**−**01**
rs531163	11	67951072	1.65E−02	LRP5	+	+	2.34E−01	1.66E−01
rs608343	11	67953406	1.82E−02	LRP5	+	+	2.34E−01	1.66E−01
rs3736228	11	67957871	1.94E−02	LRP5	−	+	2.34E−01	1.66E−01
rs624003	11	67987816	1.25E−02	PPP6R3	−	+	**2.37E**−**02**	**3.54E**−**02**
rs11822059	11	68057398	1.09E−02	PPP6R3	+	+	**2.37E**−**02**	**3.54E**−**02**
rs2840367	11	68058677	1.37E−02	PPP6R3	+	+	**2.37E**−**02**	**3.54E**−**02**
rs10501398	11	68088291	1.11E−02	PPP6R3	−	+	**2.37E**−**02**	**3.54E**−**02**
rs7108376	11	68104446	7.16E−03	PPP6R3	−	+	**2.37E**−**02**	**3.54E**−**02**
rs3740628	11	68137991	1.12E−02	PPP6R3	+	+	**2.37E**−**02**	**3.54E**−**02**
rs7128942	11	68144560	4.94E−03	PPP6R3/GAL	−	+	**2.37E**−**02**	**3.54E**−**02**/2.68E−01
rs2806277	13	99176470	6.67E−03	CLYBL	−	−	**5.45E**−**05**	2.80E−01
rs1105576	13	99178430	4.49E−03	CLYBL	−	−	**5.45E**−**05**	2.80E−01

SNP, single nucleotide polymorphisms; Chr, chromosome; Position, chromosome position; ccFDR, conjunction conditional false discovery rate; +, positive effect; −, negative effect; ALM, appendicular lean mass; BMD, bone mineral density; p.ALM, p values of gene expression validation analysis on vastus lateralis muscle biopsies; p.BMD, p value of gene expression validation analysis on iliac bone biopsies; None, not detected. Bold p values are those that are nominally significant (p <0.05).

### Novel Potential ALM-Associated SNPs Identified by cFDR

In this study, a total of 156 potential ALM-associated SNPs mapping to 15 chromosomes were identified by cFDR (**Supplementary Figure 2** and **Supplementary Table 3**). We compared the SNPs found to be significant by cFDR with previous GWAS findings (**Supplementary Table 4**) and the latest findings by Pei et al. (10). Eight LM-associated SNPs (rs12284933, rs12741884, rs3765350, rs6591341, rs6726821, rs754388, rs917727 and rs9525638) reported by previous GWASs were replicated by cFDR (SNPs marked * in **Supplementary Table 3**), 66 ALM SNPs identified as significant by cFDR (SNPs marked ** in **Supplementary Table 3**) exhibited high LD with previously-confirmed LM-associated SNPs. These 74 SNPs were considered replicates of previous GWAS findings. In total, 82 novel potential SNPs for ALM were identified by cFDR (SNPs marked *** in **Supplementary Table 3**). Of the 82 novel SNPs, 11 were located in *CPED1*, nine in *LRP5*, nine near *BMP2* and *FUSIP1P2*, six in or near *PPP6R3* and six SNPs in or near *WNT4*. Genetic variants in or near *CPED1*, *PPP6R3* and *WNT4* have already been reported by previous GWASs to be associated with LM (**Supplementary Table 3**).

### Gene Expression Validation Analysis for Potential Pleiotropic Genes and Potential ALM-Associated Genes

For the novel potential pleiotropic genes associated with both ALM and BMD, mRNA transcripts of four genes (*PPP6R3, RIN3, CLIC5* and *CLYBL*) annotated by 13 novel potential pleiotropic SNPs expressed differentially on vastus lateralis muscle between young and old adults (Bond p.ALM value in the rightmost columns of **Table 1**). mRNA transcripts of four genes on iliac bone (*PPP6R3*, *CLIC5*, *RUNX2* and *MUS81*) annotated by 17 novel potential pleiotropic SNPs were associated with whole body BMD (Bond p.BMD value in the rightmost columns of **Table 1**). *PPP6R3* and *CLIC5* were partially validated in both muscle and bone biopsies. For genes corresponding to novel potential ALM-associated SNPs, the mRNA level differences on vastus lateralis muscle between young and old adults were calculated and the p values were presented in the rightmost column of **Table 2** and **Supplementary Table 3**. mRNA transcripts level of seven genes (*PPP6R3*, *CLIC5*, *TNFSF11*, *RIN3*, *CLYBL*, *SMOC2* and *CDC42*) annotated by 18 novel potential ALM-associated SNPs expressed differentially on vastus lateralis muscle between young and compared with old adults (p value <0.05). This result suggests that these seven genes may partially participate in the aging of muscle.

**Table 2:** Top 20 novel potential ALM-associated SNPs identified by cFDR.

SNP	Chr	Position	cFDR	Mapped Gene	Effect	Gene Expression validation (p.ALM)
rs10227474	7	6525728	3.57E−04	CPED1	+	2.99E−01
rs10480747	6	7077738	2.02E−03	RUNX2/CLIC5	+	1.34E−01/**7.55E**−**06**
rs11131790	1	22520998	4.58E−04	WNT4	+	1.53E−01
rs1159531	11	35816526	3.69E−03	PPP6R3	+	**2.37E**−**02**
rs11822059	11	42038102	1.20E−03	LRP5	+	2.34E−01
rs12673968	7	42072100	3.73E−04	CPED1	−	2.99E−01
rs1414660	1	45724421	9.27E−04	LOC729796/WNT4	−	None/1.53E−01
rs1475385	11	45727343	8.56E−04	LRP5	−	2.34E−01
rs2306862	14	67957871	1.48E−03	RIN3	+	**6.10E**−**03**
rs2347227	11	67962154	2.59E−03	PPP6R3	−	**2.37E**−**02**
rs2707520	13	68055088	1.64E−03	CLYBL	−	**5.45E**−**05**
rs389700	7	68092054	3.28E−04	CPED1	+	2.99E−01
rs435260	11	68104446	2.90E−03	PPP6R3	+	**2.37E**−**02**
rs4731006	13	68123769	5.59E−04	CLYBL	−	**5.45E**−**05**
rs556442	7	68138183	4.31E−04	CPED1	+	2.99E−01
rs6038724	6	92156671	1.12E−03	RUNX2/CLIC5	−	1.34E−01/**7.55E**−**06**
rs6709795	11	120609622	4.01E−03	PPP6R3	−	**2.37E**−**02**
rs6954757	7	120660674	3.38E−09	WNT16	−	2.99E−01
rs9472536	1	166291490	3.09E−03	CDC42	−	**3.38E**−**02**
rs9935683	6	238659259	1.84E−03	RUNX2/CLIC5	−	1.34E−01/**7.55E**−**06**

SNP, single nucleotide polymorphisms; Chr, chromosome; Position, chromosome position; ccFDR, conjunction conditional false discovery rate; +, positive effect; −, negative effect; ALM, appendicular lean mass; BMD, bone mineral density; p.ALM, p values of gene expression validation analysis on human vastus lateralis muscle biopsies; None, not detected. Bold p values represent nominally significant (p <0.05).

### Functional Enrichment Analysis of Potential Pleiotropic Genes and Potential ALM-Associated Genes

As shown in **Supplementary Table 5**, genes corresponding to novel, potentially pleiotropic SNPs were enriched in GO terms for factors that play an important role in muscle development and/or bone metabolism. For example, “osteoblast development” regulates bone formation, “regulation of hormone metabolic process” participates in both muscle development (29) and bone metabolism (30). No KEGG canonical pathway was found for the novel potentially pleiotropic genes, maybe because of the small number of genes that were found. Genes corresponding to novel potentially ALM-associated SNPs were found to be enriched in 10 GO terms (**Table 3**), of which “regulation of glucocorticoid metabolic process” possessed the lowest adjusted p value. Glucocorticoid participates in the process of ALM development and could induce muscle cell atrophy (31). The results of KEGG enrichment analysis for novel potentially ALM-associated genes are presented in **Table 4**. In total, 10 KEGG pathways were enriched in, and the majority related to, cancer of different tissues. Cancer occurs in numerous tissues, including muscle, for example, rhabdomyosarcoma and leiomyosarcoma.

**Table 3:** GO Terms of genes corresponding to novel potential ALM-associated SNPs (adjP <0.05).

GO Term	GO ID	adjP	Genes
regulation of glucocorticoid metabolic process	31943	1.00E−04	BMP2, GAL, WNT4
organ morphogenesis	9887	2.00E−04	BMP2, VEGFC, WNT4, TNFSF11, RUNX2, CLIC5, CDC42, LRP5, WNT16
epithelium development	60429	2.00E−04	BMP2, VEGFC, WNT4, TNFSF11, CDC42, LRP5, WNT16, DACT2
regulation of hormone metabolic process	32350	5.00E−04	BMP2, GAL, WNT4
glucocorticoid metabolic process	8211	5.00E−04	BMP2, GAL, WNT4,
regulation of osteoblast differentiation	45667	7.00E−04	BMP2, LRP5, WNT4, RUNX2
tissue development	9888	7.00E−04	BMP2, VEGFC, WNT4, TNFSF11, RUNX2, CDC42, PRKCQ, LRP5, WNT16, DACT2
thyroid-stimulating hormone-secreting cell differentiation	60129	7.00E−04	BMP2, WNT4
receptor binding	5102	3.70E−03	BMP2, VEGFC, GAL, WNT4, TNFSF11, CDC42, NXPH1, FAM3C, WNT16
binding	5488	3.43E−02	NOL4, VEGFC, GAL, NUDT8, RUNX2, CLIC5, CLYBL, TBX10, LRP5, PRKCQ, SMOC2, DACT2, FMN2, ZNF804A, BMP2, GALNT3, WNT4, TNFSF11, CDC42, MUS81, NEIL3, NXPH1, RIN3, WNT16

GO, gene ontology term; adjP, p value adjusted by the multiple test adjustment.

**Table 4:** KEGG pathways of genes corresponding to novel potential ALM-associated SNPs (adjP <0.05).

KEGG pathway	KEGG ID	adjP	Genes
Pathways in cancer	5200	2.24E−05	BMP2, VEGFC, WNT16, WNT4, CDC42
Basal cell carcinoma	5217	2.43E−05	BMP2, WNT16, WNT4
Hedgehog signaling pathway	4340	2.43E−05	BMP2, WNT16, WNT4
Wnt signaling pathway	4310	3.00E−04	LRP5, WNT16, WNT4
Cytokine-cytokine receptor interaction	4060	1.50E−03	BMP2, VEGFC, TNFSF11
Pancreatic cancer	5212	1.60E−03	VEGFC, CDC42
Renal cell carcinoma	5211	1.60E−03	VEGFC, CDC42
Melanogenesis	4916	2.70E−03	WNT16, WNT4
T cell receptor signaling pathway	4660	2.80E−03	PRKCQ, CDC42
Tight junction	4530	3.60E−03	PRKCQ, CDC42

adjP, p value adjusted by the multiple test adjustment.

### Protein–Protein Interaction Analysis for Potential ALM-Associated Genes

**Figure 2:**
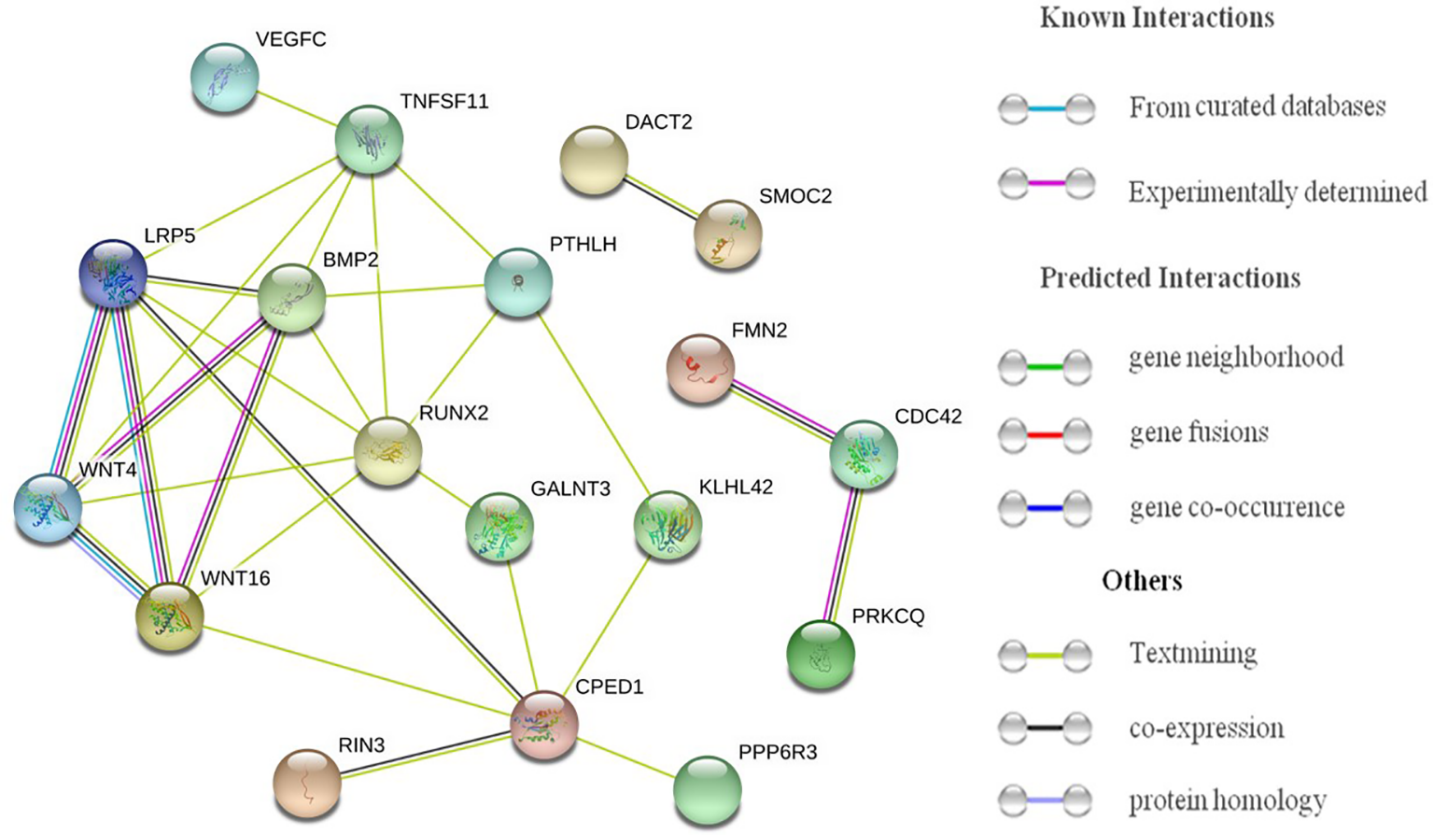
Plot of protein–protein interactions for genes corresponding to potential ALM-associated SNPs.

As shown in **Figure 2**, multiple interactions were observed among proteins produced by genes corresponding to potential ALM-associated SNPs. *LRP5*, *CPED1*, *BMP2*, *PPP6R3* and *WNT4* had at least five novel potential ALM-associated SNPs, proteins produced by them all exhibited direct or indirect interactions with proteins produced by genes corresponding to other potential ALM-associated SNPs, such as *WNT16*, *TNFSF11* and *RUNX2*. This suggests that these genes operate in a coordinated fashion in their functioning and significance for ALM. As reported in previous studies, *LRP5* is a co-receptor of Wnt proteins and is able to transduce signals through the canonical Wnt pathway (32). *CPED1* is located near *WNT16.* It has been reported that SNPs in *WNT16* regulate *CPED1* expression during lung development (33). The concentration of protein produced by *BMP2* could influence myogenesis in mesenchymal cells (34), in addition to *BMP2* also being a BMD-associated gene that plays a key role in bone formation.

## Discussion

In this study, we implemented cFDR methodology on the summary statistics of a large, independent GWAS of two highly-correlated phenotypes—ALM and BMD, successfully identifying 55 potential pleiotropic SNPs related to the two traits. Of these, 29 had successfully replicated results of previous GWAS findings, with 26 totally novel potential pleiotropic SNPs, identified by ccFDR. The mRNA transcripts of two genes (*PPP6R3* and *CLIC5*) annotated by 11 novel potential pleiotropic SNPs were partially validated in both bone and muscle gene expression validation analysis. Furthermore, by leveraging the power of pleiotropy with BMD, we identified 156 potential ALM-associated SNPs without the recruitment of any additional participants. Of the 156 potential ALM-associated SNPs, 74 have been identified in previous LM GWASs or with high LD in previous LM GWAS findings. Eighty two SNPs were novel potential ALM-associated SNPs identified by cFDR. Seven genes annotated by 18 novel potential ALM-associated SNPs were partially validated in muscle gene expression analysis. Genes found to be significant by ccFDR and/or cFDR were enriched in GO terms and/or KEGG pathways related to muscle development and/or bone metabolism. The present study demonstrated the reliability and enhanced power of cFDR methodology by detecting potential pleiotropic variants in addition to trait-specific variants for complex traits and diseases.

In pleiotropic variant analysis, *LRP5* was found to be the gene with the highest number of novel potential pleiotropic SNPs (8 SNPs mapped in total). *LRP5* is located on chromosome 11 and plays a key role in bone metabolism. It was confirmed in a large GWAS that *LRP5* is associated with BMD (35). In mice, mutations in *LRP5* were found to be associated with low BMD and abnormal retinal vasculature (36), while in humans, mutations in *LRP5* were found to cause disorders characterized by high BMD and osteoporosis–pseudoglioma syndrome, causing severe osteoporosis and blindness (37). In addition to effects on bone, *LRP5* also participates in muscle development. As a co-receptor of Wnt proteins, *LRP5* transduces Wnt signaling and regulates the steps in the process of myogenesis, such as myoblast fusion and cell proliferation (32). Given that the highest number of novel potential pleiotropic SNPs was associated with *LRP5* in ccFDR analysis, combined with its confirmed role in myogenesis and bone metabolism, we speculate that *LRP5* may be a pleiotropic gene for ALM and BMD. Additional experiments should focus on this gene to explore the underlying mechanism of pleiotropy between the two traits.

Besides *LRP5*, *PPP6R3* is also noteworthy as it is annotated by seven novel potential pleiotropic SNPs. It is one of two genes partially validated in both muscle and bone gene expression analysis. *PPP6R3* is located on chromosome 11 and can modulate the activity of protein phosphatase catalytic subunits. High expression of transcript of *PPP6R3* has been detected in skeletal muscle (38) and bone (39), and the SNPs of *PPP6R3* have already been reported in previous GWAS analyses to have pleiotropic effects on LM and BMD in the pediatric population (17). However, we still know little about the exact mechanism of how it affects muscle development or bone metabolism. Additional research is required to investigate its function in the pleiotropy of ALM and BMD.

By leveraging the power of pleiotropy with BMD, we identified 82 novel potential ALM-associated SNPs. *CPED1* is a gene annotated by 11 of them. Variants near *CPED1* and *WNT16* have been reported to have pleiotropic effects on LM and BMD in the pediatric population (17), and it has been reported that in Duchenne Muscular Dystrophy patients, up-regulation of *CPED1* by muscle-specific microRNAs improved skeletal muscle function (40). In addition to *CPED1*, *BMP2* and *LRP5* are each annotated by nine novel potential ALM-associated SNPs as found in cFDR analysis. High concentrations of *BMP2* protein can influence myogenesis, osteogenesis and adipogenesis in mesenchymal cells (34), and thus participates in muscle development. As for *LRP5*, it is a co-receptor of Wnt proteins and regulates the process of myogenesis (32). Six novel potential ALM-associated SNPs are located in or close to *WNT4* and two are located in or near *WNT16*. In KEGG canonical pathway analysis, *WNT4*, *WNT16* and *LRP5* were found to be enriched in the Wnt signaling pathway. Studies have demonstrated that Wnt ligands participate in the process of myogenesis, with both canonical and noncanonical Wnt pathways involved in the regulation of muscle formation and body tissue homeostasis (15). Four novel potential ALM-associated SNPs are located close to *RUNX2* and *CLIC5*. *RUNX2* is an important determinant of osteoblast differentiation, participating in bone formation and regulating bone metabolism (41). Though *RUNX2* is expressed on smooth muscle cells and regulates arterial calcification (42), no relationship between *RUNX2* and skeletal muscle has as yet been reported.

SNPs close to *CLIC5* have been reported to be associated with heel BMD in a previous GWAS (43). In this gene expression validation analysis, levels of mRNA transcripts of *CLIC5* were expressed differentially on vastus lateralis muscle in young compared to old adults, indicating that *CLIC5* probably participates in the ageing process of skeletal muscle. Given the high number of novel potentially ALM-associated SNPs these genes possess, combined with the evidence presented above, we hypothesize that *CPED1*, *BMP2*, *LRP5*, *WNT4*, *WNT16*, *PPP6R3*, *RUNX2* and *CLIC5* may participate in ALM development and are potential ALM-associated genes. Additional studies that replicate these findings could help to establish the validity of these observations.

In addition to total body less head BMD, we also applied the cFDR methodology to the same ALM GWAS data and eBMD GWAS data released from the UK Biobank in 2018 (n = 426,824). The UK biobank replicates many previous BMD GWAS findings. But in the first enrichment analysis (QQ plot), no clear pleiotropic enrichment was found between the two traits. The 2018 eBMD data was estimated from quantitative heel ultrasound measurements. Different skeleton sites do not have the same genetic determination. The degree of pleiotropy in ALM may also be different (44).

Although we succeeded in identifying potential pleiotropic variants for ALM and BMD in addition to novel potential variants for ALM, there were some limitations to our study. Firstly, as empirical research, our analysis aimed to provide a greater number of novel potential pleiotropic or trait-specific SNPs/genes for ALM and/or BMD. Variants identified by cFDR and ccFDR still require to be replicated and/or functional validation research conducted to be robustly confirmed. Secondly, we were not able to obtain raw genotype data and so only analyzed the summary statistics of the GWAS. The contribution of our present findings to the proportion of variability in ALM could not be estimated. Thirdly, we were not able to acquire the bone gene expression profiling data of children, so data from postmenopausal women were used for BMD gene expression analysis. This population is older than children and represents a single sex, thus affecting the results slightly. Lastly, the ALM dataset (n = 73,420) is larger than the BMD dataset (n = 10,414), the effect of imbalanced sample size on the cFDR results was not discussed in the original methodology development paper, it is worth further theoretical investigation, but that is not we can do in the present empirical study.

In conclusion, the cFDR approach increased the effective sample size of existing GWAS data and enhanced the detection of potential genetic variants. By leveraging pleiotropy with correlations in BMD phenotype, combined with the use of the cFDR methodology, we identified 26 novel potential pleiotropic SNPs associated with ALM and BMD, with 82 novel potential ALM-associated SNPs. Genes encompassed by these SNPs play an important role in muscle development and/or bone metabolism. Our findings shed novel insight into the genetic variability of ALM in addition to the shared genetic basis underlying ALM and BMD.

## Data Availability Statement

Publicly available datasets were analyzed in this study. The data for appendicular lean mass can be found at: http://www.gefos.org/?q=content/adult-lean-mass-gwas-2017, and data for BMD can be found at: http://www.gefos.org/?q=content/data-release-2017.

## Author Contributions

H-LL conceived and initiated the development of this study; she is responsible for general development and design of the study and contributed to critical revisions and finalization of the manuscript; she is a guarantor. CP contributed to the acquisition and analysis of the data and drafted the manuscript. H-WD contributed to the study design and critical revisions. K-JS, XL, Y-QS, S-DH, Q-CC, W-XL, and H-HY contributed to data analysis. FL, JS, and C-PZ contributed to the general study design and development. All authors have given approval to the final version of the manuscript. All authors agree to be accountable for the work and ensure that any questions relating to the accuracy and integrity of the paper are investigated and properly resolved.

## Funding

H-LL, CP, and FL were supported by Guangzhou Planed Project of Science and Technology, Guangzhou, China [201903010091, 201704020105], and Medical Science and Technology Foundation of Guangdong Province (CN), [A2019005]. H-WD was partially supported by grants from the National Institutes of Health [AR069055, U19 AG055373, R01 MH104680, R01AR059781 and P20GM109036] and the Edward G. Schlieder Endowment Fund to Tulane University. S-DH and JS were supported by China National Natural Scientific Foundation [81800794, 81770878]] and Natural Science Foundation of Guangdong province doctor initiated (vertical collaboration) project [2017A030310390].

## Conflict of Interest

The authors declare that the research was conducted in the absence of any commercial or financial relationships that could be construed as a potential conflict of interest.
