# Supplementary material for: Enhanced Identification of Novel Potential Variants for Appendicular Lean Mass by Leveraging Pleiotropy With Bone Mineral Density
Source: Front Immunol. 2021 Apr 6;12:643894. doi: 10.3389/fimmu.2021.643894 (PMC8056257; doi:10.3389/fimmu.2021.643894)

Supplementary Figure 1 Manhattan plot of conjunction $-\log_{10}$ ccFDR values for potential pleiotropic SNPs. The red line marking the conditional $-\log_{10}$ ccFDR value of 1.3 corresponds to ccFDR < 0.05. The figure represents genomic locations of potential pleiotropic SNPs associated with both ALM and BMD. Details of the potential pleiotropic SNPs are listed in Supplementary Table 1.

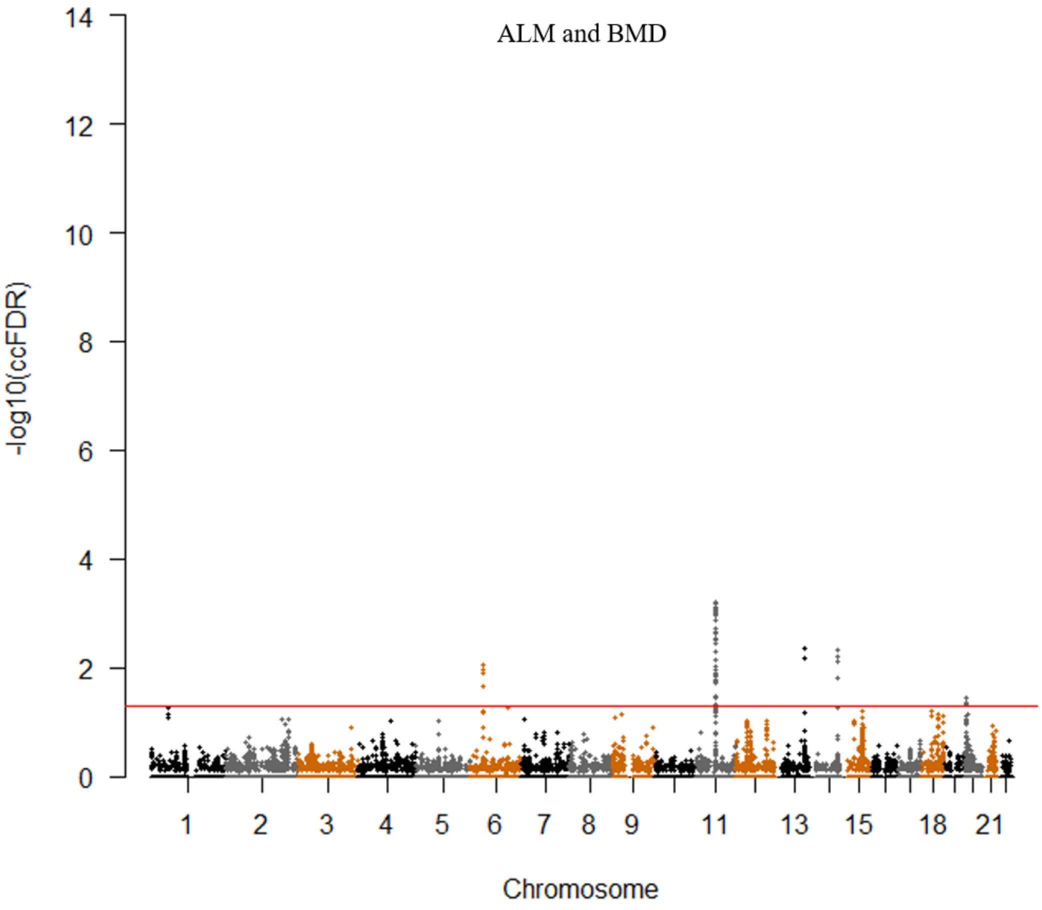

Supplement: Supplementary file 1 [file Image_1.pdf]
