# Supplementary material for: Enhanced Identification of Novel Potential Variants for Appendicular Lean Mass by Leveraging Pleiotropy With Bone Mineral Density
Source: Front Immunol. 2021 Apr 6;12:643894. doi: 10.3389/fimmu.2021.643894 (PMC8056257; doi:10.3389/fimmu.2021.643894)

Supplementary Figure 2 Manhattan plot of conditional $-\log_{10}$ cFDR values for ALM given BMD (ALM|BMD). The red line marking the conditional $-\log_{10}$ cFDR value of 1.3 corresponds to cFDR < 0.05. The figure presents the chromosomal locations of cFDR-significant SNPs potentially associated with ALM.

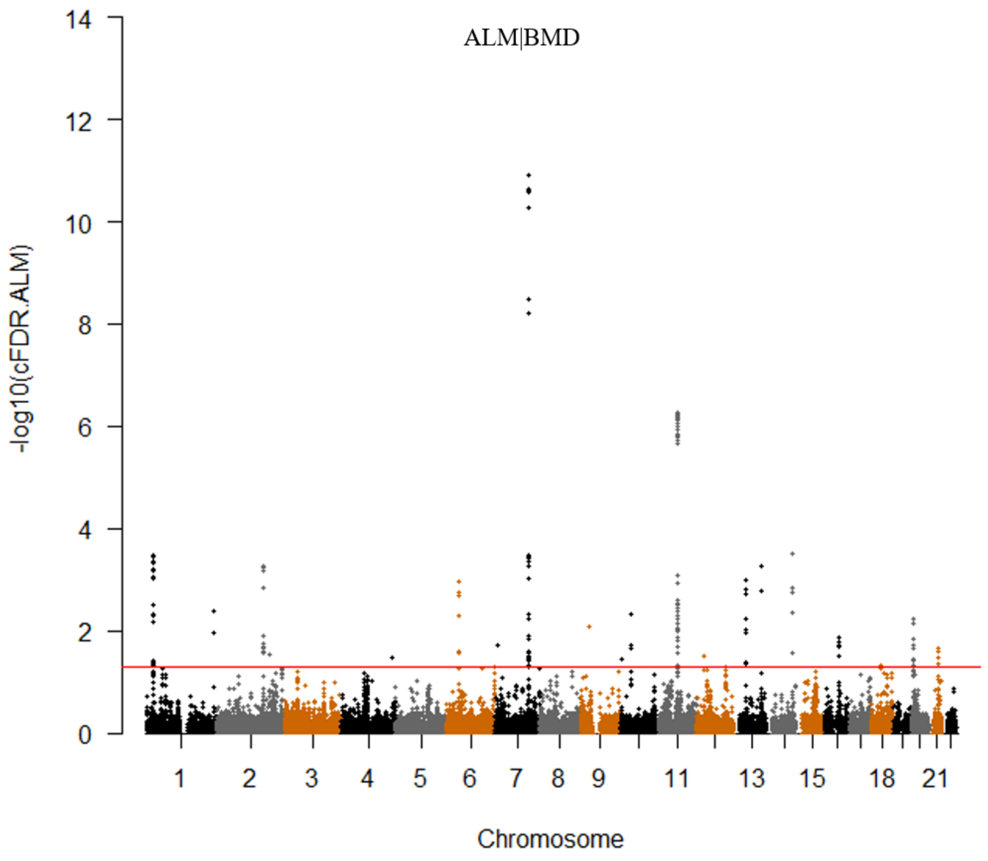

Supplement: Supplementary file 2 [file Image_2.pdf]
