# Supplementary material for: Enhanced Identification of Novel Potential Variants for Appendicular Lean Mass by Leveraging Pleiotropy With Bone Mineral Density
Source: Front Immunol. 2021 Apr 6;12:643894. doi: 10.3389/fimmu.2021.643894 (PMC8056257; doi:10.3389/fimmu.2021.643894)
Supplement: Supplementary file 3 [file Table_1.docx]

**Supplementary Table 1 Potential pleiotropic SNPs for ALM and BMD identified by ccFDR**

| SNP | Chr | Position | ccFDR | Mapped Gene | Effect | | Gene Expressional validation | | |
| --- | --- | --- | --- | --- | --- | --- | --- | --- | --- |
|  |  |  |  |  | ALM | BMD | | p.ALM | p.BMD |
| ^***^rs1159531 | 20 | 7051595 | 3.53E-02 | BMP2/FUSIP1P2 | + | + | | 1.48E-01/None | 7.96E-02/None |
| ^***^rs1159530 | 20 | 7051709 | 4.41E-02 | BMP2/FUSIP1P2 | + | + | | 1.48E-01/None | 7.96E-02/None |
| ^***^rs1884303 | 20 | 7054353 | 4.72E-02 | BMP2/FUSIP1P2 | - | + | | 1.48E-01/None | 7.96E-02/None |
| ^***^rs9472536 | 6 | 45724421 | 1.08E-02 | RUNX2/CLIC5 | - | + | | 1.34E-01/**7.55E-06** | **1.31E-02/2.39E-03** |
| ^***^rs6923368 | 6 | 45727343 | 8.66E-03 | RUNX2/CLIC5 | - | + | | 1.34E-01/**7.55E-06** | **1.31E-02/2.39E-03** |
| ^***^rs16873645 | 6 | 45773425 | 1.22E-02 | RUNX2/CLIC5 | + | + | | 1.34E-01/**7.55E-06** | **1.31E-02/2.39E-03** |
| ^***^rs12333018 | 6 | 45782629 | 2.16E-02 | RUNX2/CLIC5 | + | + | | 1.34E-01/**7.55E-06** | **1.31E-02/2.39E-03** |
| ^***^rs648732 | 11 | 65387743 | 4.83E-02 | MUS81 | - | + | | 9.53E-01 | **2.88E-02** |
| ^***^rs3758938 | 11 | 67158938 | 3.50E-02 | TBX10 | - | - | | 4.14E-01 | 7.68E-01 |
| ^***^rs2306862 | 11 | 67934086 | 3.19E-03 | LRP5 | - | + | | 2.34E-01 | 1.66E-01 |
| ^***^rs314750 | 11 | 67938604 | 9.83E-03 | LRP5 | + | + | | 2.34E-01 | 1.66E-01 |
| ^***^rs923346 | 11 | 67938951 | 3.60E-03 | LRP5 | + | + | | 2.34E-01 | 1.66E-01 |
| ^***^rs599083 | 11 | 67948922 | 1.46E-02 | LRP5 | + | + | | 2.34E-01 | 1.66E-01 |
| ^***^rs556442 | 11 | 67949266 | 3.26E-02 | LRP5 | + | + | | 2.34E-01 | **1.66E-01** |
| ^***^rs531163 | 11 | 67951072 | 1.65E-02 | LRP5 | + | + | | 2.34E-01 | 1.66E-01 |
| ^***^rs608343 | 11 | 67953406 | 1.82E-02 | LRP5 | + | + | | 2.34E-01 | 1.66E-01 |
| ^***^rs3736228 | 11 | 67957871 | 1.94E-02 | LRP5 | - | + | | 2.34E-01 | 1.66E-01 |
| ^**^rs901823 | 11 | 67962154 | 1.99E-02 | LRP5 | + | + | | 2.34E-01 | 1.66E-01 |
| ^***^rs624003 | 11 | 67987816 | 1.25E-02 | PPP6R3 | - | + | | **2.37E-02** | **3.54E-02** |
| ^**^rs11228258 | 11 | 68010904 | 7.77E-04 | PPP6R3 | - | + | | **2.37E-02** | **3.54E-02** |
| ^**^rs11228269 | 11 | 68046372 | 7.77E-04 | PPP6R3 | + | + | | **2.37E-02** | **3.54E-02** |
| ^**^rs7925275 | 11 | 68047009 | 9.42E-04 | PPP6R3 | + | + | | **2.37E-02** | **3.54E-02** |
| ^**^rs7106259 | 11 | 68055088 | 1.08E-03 | PPP6R3 | + | + | | **2.37E-02** | **3.54E-02** |
| ^***^rs11822059 | 11 | 68057398 | 1.09E-02 | PPP6R3 | + | + | | **2.37E-02** | **3.54E-02** |
| ^***^rs2840367 | 11 | 68058677 | 1.37E-02 | PPP6R3 | + | + | | **2.37E-02** | **3.54E-02** |
| ^**^rs12281742 | 11 | 68060205 | 6.05E-04 | PPP6R3 | + | + | | **2.37E-02** | **3.54E-02** |
| ^**^rs4316515 | 11 | 68065416 | 1.04E-03 | PPP6R3 | + | + | | **2.37E-02** | **3.54E-02** |
| ^**^rs7944870 | 11 | 68065684 | 1.36E-03 | PPP6R3 | + | + | | **2.37E-02** | **3.54E-02** |
| ^**^rs948315 | 11 | 68065716 | 7.90E-04 | PPP6R3 | + | + | | **2.37E-02** | **3.54E-02** |
| ^*^rs12284933 | 11 | 68076065 | 2.87E-03 | PPP6R3 | - | + | | **2.37E-02** | **3.54E-02** |
| ^**^rs12271290 | 11 | 68082812 | 6.53E-04 | PPP6R3 | - | + | | **2.37E-02** | **3.54E-02** |
| ^**^rs7102898 | 11 | 68085446 | 1.94E-03 | PPP6R3 | - | + | | **2.37E-02** | **3.54E-02** |
| ^**^rs7119422 | 11 | 68087730 | 9.67E-03 | PPP6R3 | + | + | | **2.37E-02** | **3.54E-02** |
| ^***^rs10501398 | 11 | 68088291 | 1.11E-02 | PPP6R3 | - | + | | **2.37E-02** | **3.54E-02** |
| ^**^rs7109294 | 11 | 68088669 | 1.12E-03 | PPP6R3 | + | + | | **2.37E-02** | **3.54E-02** |
| ^**^rs6591341 | 11 | 68092054 | 6.87E-04 | PPP6R3 | - | + | | **2.37E-02** | **3.54E-02** |
| ^**^rs3740631 | 11 | 68098298 | 2.22E-03 | PPP6R3 | + | + | | **2.37E-02** | **3.54E-02** |
| ^***^rs7108376 | 11 | 68104446 | 7.16E-03 | PPP6R3 | - | + | | **2.37E-02** | **3.54E-02** |
| ^**^rs10896347 | 11 | 68109703 | 2.30E-03 | PPP6R3 | - | + | | **2.37E-02** | **3.54E-02** |
| ^**^rs10896348 | 11 | 68113944 | 8.84E-04 | PPP6R3 | + | + | | **2.37E-02** | **3.54E-02** |
| ^**^rs7118897 | 11 | 68117256 | 8.30E-04 | PPP6R3 | - | + | | **2.37E-02** | **3.54E-02** |
| ^**^rs3758643 | 11 | 68123769 | 9.13E-04 | PPP6R3 | - | + | | **2.37E-02** | **3.54E-02** |
| ^**^rs12283755 | 11 | 68128234 | 1.04E-03 | PPP6R3 | + | + | | **2.37E-02** | **3.54E-02** |
| ^**^rs7104877 | 11 | 68134178 | 7.55E-04 | PPP6R3 | + | + | | **2.37E-02** | **3.54E-02** |
| ^**^rs2236708 | 11 | 68134621 | 7.77E-04 | PPP6R3 | - | + | | **2.37E-02** | **3.54E-02** |
| ^**^rs11228292 | 11 | 68136348 | 8.14E-04 | PPP6R3 | - | + | | **2.37E-02** | **3.54E-02** |
| ^***^rs3740628 | 11 | 68137991 | 1.12E-02 | PPP6R3 | + | + | | **2.37E-02** | **3.54E-02** |
| ^**^rs4988291 | 11 | 68138183 | 1.08E-03 | PPP6R3 | - | + | | **2.37E-02** | **3.54E-02** |
| ^***^rs7128942 | 11 | 68144560 | 4.94E-03 | PPP6R3/GAL | - | + | | **2.37E-02** | **3.54E-02**/2.68E-01 |
| ^**^rs8010344 | 14 | 92156671 | 1.52E-02 | RIN3 | - | + | | **6.10E-03** | 3.42E-01 |
| ^**^rs7146689 | 14 | 92169777 | 7.70E-03 | RIN3 | - | + | | **6.10E-03** | 3.42E-01 |
| ^**^rs10498635 | 14 | 92173062 | 6.47E-03 | RIN3 | + | + | | **6.10E-03** | 3.42E-01 |
| ^*^rs754388 | 14 | 92185163 | 4.65E-03 | RIN3 | - | + | | **6.10E-03** | 3.42E-01 |
| ^***^rs2806277 | 13 | 99176470 | 6.67E-03 | CLYBL | - | - | | **5.45E-05** | 2.80E-01 |
| ^***^rs1105576 | 13 | 99178430 | 4.49E-03 | CLYBL | - | - | | **5.45E-05** | 2.80E-01 |

Column definition: SNP – single nucleotide polymorphisms; SNPs with ^*^ were reported by previous GWASs to be pleiotropic SNPs associated with both lean mass and BMD; SNPs with ^**^ reside in the same LD block as previously reported pleiotropic SNPs associated with lean mass and BMD; SNPs with ^***^ were novel potential pleiotropic SNPs identified by ccFDR; Chr – chromosome; Position – chromosome position; ccFDR – conjunction conditional false discovery rate; + – positive effect; - – negative effect; ALM – appendicular lean mass; BMD – bone mineral density; p.ALM – p values of gene expressional validation analysis on vastus lateralis muscle biopsies; p.BMD – p value of gene expression validation analysis on iliac bone biopsies; None – not detected. Bold p values are those that are nominally significant (p < 0.05).
