# Supplementary material for: Enhanced Identification of Novel Potential Variants for Appendicular Lean Mass by Leveraging Pleiotropy With Bone Mineral Density
Source: Front Immunol. 2021 Apr 6;12:643894. doi: 10.3389/fimmu.2021.643894 (PMC8056257; doi:10.3389/fimmu.2021.643894)
Supplement: Supplementary file 4 [file Table_2.docx]

**Supplementary Table 2 Potential pleiotropic SNPs for lean mass and BMD reported by previous GWAS analysis( https://www.ebi.ac.uk/gwas, Jan 2020).**

| Study | SNPs | Mapped Gene | Mapped Trait |
| --- | --- | --- | --- |
| Bivariate genome-wide association meta-analysis of pediatric musculoskeletal traits reveals pleiotropic effects at the SREBF1/TOM1L2 locus. | rs3765350 | WNT4 | Bone density and lean body mass |
| Bivariate genome-wide association meta-analysis of pediatric musculoskeletal traits reveals pleiotropic effects at the SREBF1/TOM1L2 locus. | rs12741884 | MIR4418/LOC101060363 | Bone density and lean body mass |
| Bivariate genome-wide association meta-analysis of pediatric musculoskeletal traits reveals pleiotropic effects at the SREBF1/TOM1L2 locus. | rs6684375 | LOC101060363/LOC105376856 | Bone density and lean body mass |
| Bivariate genome-wide association meta-analysis of pediatric musculoskeletal traits reveals pleiotropic effects at the SREBF1/TOM1L2 locus. | rs6726821 | CSRNP3/GALNT3 | Bone density and lean body mass |
| Bivariate genome-wide association meta-analysis of pediatric musculoskeletal traits reveals pleiotropic effects at the SREBF1/TOM1L2 locus. | rs7672749 | MEPE/HSP90AB3P | Bone density and lean body mass |
| Bivariate genome-wide association meta-analysis of pediatric musculoskeletal traits reveals pleiotropic effects at the SREBF1/TOM1L2 locus. | rs13245690 | CPED1 | Bone density and lean body mass |
| Bivariate genome-wide association meta-analysis of pediatric musculoskeletal traits reveals pleiotropic effects at the SREBF1/TOM1L2 locus. | rs917727 | FAM3C | Bone density and lean body mass |
| Bivariate genome-wide association meta-analysis of pediatric musculoskeletal traits reveals pleiotropic effects at the SREBF1/TOM1L2 locus. | rs12284933 | PPP6R3 | bone density, lean body mass |
| Bivariate genome-wide association meta-analysis of pediatric musculoskeletal traits reveals pleiotropic effects at the SREBF1/TOM1L2 locus. | rs9525638 | LOC105370177/TNFSF11 | bone density, lean body mass |
| Bivariate genome-wide association meta-analysis of pediatric musculoskeletal traits reveals pleiotropic effects at the SREBF1/TOM1L2 locus. | rs754388 | RIN3 | bone density, lean body mass |
| Bivariate genome-wide association meta-analysis of pediatric musculoskeletal traits reveals pleiotropic effects at the SREBF1/TOM1L2 locus. | rs7501812 | TOM1L2 | bone density, lean body mass |
| Bivariate genome-wide association meta-analysis of pediatric musculoskeletal traits reveals pleiotropic effects at the SREBF1/TOM1L2 locus. | rs2955382 | GID4 | bone density, lean body mass |
