# Supplementary material for: Enhanced Identification of Novel Potential Variants for Appendicular Lean Mass by Leveraging Pleiotropy With Bone Mineral Density
Source: Front Immunol. 2021 Apr 6;12:643894. doi: 10.3389/fimmu.2021.643894 (PMC8056257; doi:10.3389/fimmu.2021.643894)
Supplement: Supplementary file 5 [file Table_3.docx]

**Supplementary Table 3. Potential ALM-associated SNPs identified by cFDR**

| SNP |  | Chr | Position | cFDR | Mapped Gene | Effect | Gene Expression validation (p.ALM) |
| --- | --- | --- | --- | --- | --- | --- | --- |
| ^***^rs6466767 |  | 7 | 6525728 | 3.57E-04 | CPED1 | + | 2.99E-01 |
| ^**^rs1357756 |  | 7 | 7051595 | 3.42E-02 | CPED1 | - | 2.99E-01 |
| ^**^rs13431319 |  | 2 | 7051709 | 1.26E-02 | GALNT3 | - | 1.13E-01 |
| ^*^rs754388 |  | 14 | 7054289 | 3.10E-04 | RIN3 | - | **6.10E-03** |
| ^**^rs7586085 |  | 2 | 7054353 | 6.54E-04 | FAM130A2/GALNT3 | + | None/1.13E-01 |
| ^***^rs16914332 |  | 9 | 7077677 | 8.59E-03 | MIRN873/LOC286239 | - | None/None |
| ^***^rs16873645 |  | 6 | 7077738 | 2.02E-03 | RUNX2/CLIC5 | + | 1.34E-01/**7.55E-06** |
| ^**^rs1534016 |  | 7 | 7119430 | 3.55E-02 | CPED1 | + | 2.99E-01 |
| ^**^rs7118897 |  | 11 | 7119612 | 6.21E-07 | PPP6R3 | - | **2.37E-02** |
| ^***^rs1833188 |  | 16 | 7120181 | 2.03E-02 | LOC642659/LOC388276 | + | None/None |
| ^**^rs10261671 |  | 7 | 8572785 | 9.41E-04 | CPED1 | - | 2.99E-01 |
| ^**^rs8010344 |  | 14 | 22287997 | 4.37E-03 | RIN3 | - | **6.10E-03** |
| ^***^rs4930461 |  | 11 | 22309033 | 1.35E-02 | LOC390213/NUDT8 | + | None/None |
| ^***^rs11131790 |  | 4 | 22319903 | 3.44E-02 | VEGFC/NEIL3 | + | 6.92E-01/4.74E-01 |
| ^**^rs7795660 |  | 7 | 22323074 | 2.48E-02 | CPED1 | - | 2.99E-01 |
| ^**^rs17536328 |  | 13 | 22345319 | 1.97E-03 | FABP3P2/TNFSF11 | - | None/**9.35E-04** |
| ^***^rs531163 |  | 11 | 22345997 | 8.91E-03 | LRP5 | + | 2.34E-01 |
| ^***^rs11698567 |  | 20 | 22365474 | 3.71E-02 | BMP2/FUSIP1P2 | + | 1.48E-01/None |
| ^**^rs3801382 |  | 7 | 22467282 | 5.39E-11 | FAM3C | + | None |
| ^***^rs2235529 |  | 1 | 22520998 | 4.58E-04 | WNT4 | + | 1.53E-01 |
| ^**^rs9525641 |  | 13 | 27874655 | 1.09E-02 | TNFSF11 | + | **9.35E-04** |
| ^**^rs953247 |  | 1 | 27874696 | 4.00E-03 | FMN2 | - | 1.13E-01 |
| ^**^rs7109294 |  | 11 | 29116734 | 6.94E-07 | PPP6R3 | + | **2.37E-02** |
| ^***^rs440544 |  | 21 | 29949102 | 2.50E-02 | RPL34P3/LOC266693 | + | None/None |
| ^**^rs7925275 |  | 11 | 35794508 | 5.93E-07 | PPP6R3 | + | **2.37E-02** |
| ^***^rs11822059 |  | 11 | 35816526 | 3.69E-03 | PPP6R3 | + | **2.37E-02** |
| ^***^rs12042083 |  | 1 | 35817449 | 4.88E-03 | WNT4/LOC343384 | + | 1.53E-01/None |
| ^***^rs11686403 |  | 2 | 35819783 | 1.79E-02 | GALNT3 | - | 1.13E-01 |
| ^**^rs6950680 |  | 7 | 36172566 | 5.60E-04 | CPED1 | + | 2.99E-01 |
| ^**^rs6952113 |  | 7 | 36174115 | 5.62E-04 | CPED1 | - | 2.99E-01 |
| ^**^rs7944870 |  | 11 | 36201445 | 1.14E-06 | PPP6R3 | + | **2.37E-02** |
| ^**^rs7119422 |  | 11 | 42009354 | 3.10E-03 | PPP6R3 | + | **2.37E-02** |
| ^***^rs901823 |  | 11 | 42021925 | 9.55E-03 | LRP5 | + | 2.34E-01 |
| ^***^rs7918373 |  | 10 | 42026577 | 3.48E-02 | PRKCQ | - | 6.92E-01 |
| ^***^rs923346 |  | 11 | 42038102 | 1.20E-03 | LRP5 | + | 2.34E-01 |
| ^**^rs7146689 |  | 14 | 42041029 | 1.76E-03 | RIN3 | - | **6.10E-03** |
| ^***^rs4731006 |  | 7 | 42043319 | 1.42E-02 | CPED1 | - | 2.99E-01 |
| ^***^rs9349325 |  | 6 | 42045671 | 2.51E-02 | RUNX2/CLIC5 | - | 1.34E-01/**7.55E-06** |
| ^***^rs7950900 |  | 11 | 42046024 | 2.02E-02 | PPP6R3 | - | **2.37E-02** |
| ^***^rs6954757 |  | 7 | 42072100 | 3.73E-04 | CPED1 | - | 2.99E-01 |
| ^***^rs7412010 |  | 1 | 45724421 | 9.27E-04 | LOC729796/WNT4 | - | None/1.53E-01 |
| ^***^rs2306862 |  | 11 | 45727343 | 8.56E-04 | LRP5 | - | 2.34E-01 |
| ^**^rs7106259 |  | 11 | 45773425 | 9.85E-07 | PPP6R3 | + | **2.37E-02** |
| ^*^rs3765350 |  | 1 | 45782629 | 3.47E-04 | WNT4 | + | 1.53E-01 |
| ^**^rs1968294 |  | 2 | 45796974 | 1.23E-02 | GALNT3 | - | 1.13E-01 |
| ^***^rs7233297 |  | 18 | 45799027 | 4.84E-02 | NOL4 | - | 6.19E-01 |
| ^***^rs9932063 |  | 16 | 50561058 | 2.05E-02 | LOC642659/LOC388276 | - | None/None |
| ^*^rs12284933 |  | 11 | 50562501 | 1.57E-06 | PPP6R3 | - | **2.37E-02** |
| ^***^rs9935683 |  | 16 | 50566574 | 1.68E-02 | LOC642659/LOC388276 | + | None/None |
| ^***^rs9931445 |  | 16 | 50568606 | 1.31E-02 | LOC642659/LOC388276 | - | None/None |
| ^**^rs718766 |  | 7 | 50577387 | 2.50E-11 | FAM3C | + | None |
| ^**^rs7325635 |  | 13 | 50581318 | 1.87E-03 | FABP3P2/TNFSF11 | - | None/**9.35E-04** |
| ^***^rs1884303 |  | 20 | 65387743 | 7.18E-03 | BMP2/FUSIP1P2 | - | 1.48E-01/None |
| ^***^rs12333018 |  | 6 | 67151471 | 5.00E-03 | RUNX2/CLIC5 | + | 1.34E-01/**7.55E-06** |
| ^***^rs3758938 |  | 11 | 67152290 | 8.96E-03 | TBX10 | - | 4.14E-01 |
| ^***^rs1110492 |  | 16 | 67158938 | 3.14E-02 | LOC642659/LOC388276 | + | None/None |
| ^*^rs9525638 |  | 13 | 67934086 | 1.02E-03 | FABP3P2/TNFSF11 | + | None/**9.35E-04** |
| ^***^rs10480747 |  | 7 | 67938604 | 3.57E-02 | CPED1 | + | 2.99E-01 |
| ^**^rs7128942 |  | 11 | 67938951 | 4.94E-03 | PPP6R3/GAL | - | **2.37E-02**/1.73E-01 |
| ^***^rs1475386 |  | 10 | 67948922 | 2.20E-02 | LOC439954/PBEF2 | - | None/None |
| ^***^rs1475385 |  | 10 | 67949266 | 1.99E-02 | LOC439954/PBEF2 | + | None/None |
| ^***^rs1414660 |  | 1 | 67951072 | 1.10E-02 | FMN2 | - | 1.13E-01 |
| ^***^rs17373665 |  | 20 | 67953406 | 3.75E-02 | BMP2/FUSIP1P2 | - | 1.48E-01/None |
| ^**^rs10498635 |  | 14 | 67957871 | 1.48E-03 | RIN3 | + | **6.10E-03** |
| ^***^rs7108376 |  | 11 | 67962154 | 2.59E-03 | PPP6R3 | - | **2.37E-02** |
| ^***^rs184887 |  | 16 | 67987816 | 1.87E-02 | LOC642659/LOC388276 | + | None/None |
| ^**^rs13232048 |  | 7 | 68010904 | 5.51E-04 | CPED1 | - | 2.99E-01 |
| ^**^rs13422985 |  | 2 | 68046372 | 2.56E-02 | GALNT3 | + | 1.13E-01 |
| ^***^rs648732 |  | 11 | 68047009 | 4.83E-02 | MUS81 | - | 9.53E-01 |
| ^***^rs6709795 |  | 2 | 68050121 | 2.83E-02 | ZNF804A/ELF2P4 | - | 5.06E-01/None |
| ^***^rs2806277 |  | 13 | 68055088 | 1.64E-03 | CLYBL | - | **5.45E-05** |
| ^**^rs17458078 |  | 13 | 68057398 | 9.80E-03 | FABP3P2/TNFSF11 | + | None/**9.35E-04** |
| ^***^rs10274324 |  | 7 | 68058677 | 3.03E-02 | CPED1 | - | 2.99E-01 |
| ^**^rs2303393 |  | 2 | 68060205 | 2.06E-02 | GALNT3 | + | 1.13E-01 |
| ^***^rs11771945 |  | 7 | 68065416 | 3.75E-02 | CPED1 | + | 2.99E-01 |
| ^***^rs6690148 |  | 1 | 68065684 | 4.08E-02 | WNT4/LOC343384 | - | 1.53E-01/None |
| ^***^rs7515106 |  | 1 | 68065716 | 6.76E-03 | WNT4/LOC343384 | - | 1.53E-01/None |
| ^**^rs3758643 |  | 11 | 68076065 | 6.88E-07 | PPP6R3 | - | **2.37E-02** |
| ^**^rs2236708 |  | 11 | 68082812 | 5.37E-07 | PPP6R3 | - | **2.37E-02** |
| ^***^rs2707520 |  | 7 | 68085446 | 4.86E-02 | CPED1/WNT16 | - | /2.99E-01 |
| ^***^rs3740628 |  | 11 | 68087730 | 4.14E-03 | PPP6R3 | + | **2.37E-02** |
| ^**^rs12185748 |  | 2 | 68088291 | 6.57E-04 | FAM130A2/GALNT3 | - | None/1.13E-01 |
| ^***^rs314750 |  | 11 | 68088669 | 5.99E-03 | LRP5 | + | 2.34E-01 |
| ^***^rs1917113 |  | 7 | 68092054 | 3.28E-04 | CPED1 | + | 2.99E-01 |
| ^***^rs10501398 |  | 11 | 68098298 | 4.24E-03 | PPP6R3 | - | **2.37E-02** |
| ^***^rs2840367 |  | 11 | 68104446 | 2.90E-03 | PPP6R3 | + | **2.37E-02** |
| ^**^rs12271290 |  | 11 | 68109703 | 8.60E-07 | PPP6R3 | - | **2.37E-02** |
| ^**^rs12281742 |  | 11 | 68113944 | 1.50E-06 | PPP6R3 | + | **2.37E-02** |
| ^***^rs3736228 |  | 11 | 68117256 | 1.55E-02 | LRP5 | - | 2.34E-01 |
| ^***^rs1105576 |  | 13 | 68123769 | 5.59E-04 | CLYBL | - | **5.45E-05** |
| ^**^rs3740631 |  | 11 | 68128234 | 1.57E-06 | PPP6R3 | + | **2.37E-02** |
| ^***^rs2763264 |  | 6 | 68134178 | 4.94E-02 | DACT2/SMOC2 | - | 3.76E-01/**1.00E-05** |
| ^**^rs7798060 |  | 7 | 68134621 | 6.05E-03 | CPED1 | - | 2.99E-01 |
| ^**^rs13427924 |  | 2 | 68136348 | 2.17E-02 | GALNT3 | + | 1.13E-01 |
| ^***^rs10500083 |  | 7 | 68137991 | 3.83E-02 | CPED1 | + | 2.99E-01 |
| ^***^rs12673968 |  | 7 | 68138183 | 4.31E-04 | CPED1 | + | 2.99E-01 |
| ^***^rs389700 |  | 21 | 68144560 | 3.31E-02 | RPL34P3/LOC266693 | - | None/None |
| ^***^rs6923368 |  | 6 | 92156671 | 1.12E-03 | RUNX2/CLIC5 | - | 1.34E-01/**7.55E-06** |
| ^***^rs3920498 |  | 1 | 92169777 | 4.72E-02 | WNT4/LOC343384 | + | 1.53E-01/None |
| ^**^rs11228269 |  | 11 | 92173062 | 7.27E-07 | PPP6R3 | + | **2.37E-02** |
| ^**^rs1404268 |  | 7 | 92180873 | 4.95E-02 | CPED1 | - | 2.99E-01 |
| ^***^rs556442 |  | 11 | 92185163 | 2.67E-02 | LRP5 | + | 2.34E-01 |
| ^***^rs10227474 |  | 7 | 99176470 | 1.86E-02 | NXPH1 | + | 6.31E-01 |
| ^**^rs12283755 |  | 11 | 99178430 | 7.59E-07 | PPP6R3 | + | **2.37E-02** |
| ^***^rs1884302 |  | 20 | 120518813 | 4.77E-02 | BMP2/FUSIP1P2 | - | 1.48E-01/None |
| ^**^rs7776725 |  | 7 | 120522298 | 1.20E-11 | FAM3C | + | None |
| ^**^rs3779381 |  | 7 | 120524294 | 6.15E-09 | WNT16 | + | 2.99E-01 |
| ^***^rs2536150 |  | 7 | 120530418 | 3.27E-02 | CPED1 | + | 2.99E-01 |
| ^**^rs3801387 |  | 7 | 120563517 | 2.60E-11 | WNT16 | + | 2.99E-01 |
| ^***^rs2347228 |  | 12 | 120564855 | 3.16E-02 | KLHDC5/PTHLH | + | None/3.85E-01 |
| ^**^rs10896347 |  | 11 | 120570787 | 8.98E-07 | PPP6R3 | - | **2.37E-02** |
| ^***^rs2347227 |  | 12 | 120577523 | 3.18E-02 | KLHDC5/PTHLH | + | None/3.85E-01 |
| ^***^rs624003 |  | 11 | 120609622 | 4.01E-03 | PPP6R3 | - | **2.37E-02** |
| ^**^rs7104877 |  | 11 | 120613474 | 8.65E-07 | PPP6R3 | + | **2.37E-02** |
| ^**^rs10085590 |  | 7 | 120619259 | 2.66E-02 | CPED1 | + | 2.99E-01 |
| ^**^rs6710388 |  | 2 | 120639429 | 5.71E-04 | FAM130A2/GALNT3 | - | None/1.13E-01 |
| ^***^rs608343 |  | 11 | 120640843 | 1.03E-02 | LRP5 | + | 2.34E-01 |
| ^**^rs6710518 |  | 2 | 120644983 | 5.83E-04 | FAM130A2/GALNT3 | - | None/1.13E-01 |
| ^**^rs6965195 |  | 7 | 120655239 | 3.47E-02 | CPED1 | - | 2.99E-01 |
| ^**^rs7102898 |  | 11 | 120660314 | 1.52E-06 | PPP6R3 | - | **2.37E-02** |
| ^***^rs2707466 |  | 7 | 120660674 | 3.38E-09 | WNT16 | - | 2.99E-01 |
| ^**^rs1554634 |  | 7 | 120665597 | 1.45E-02 | CPED1 | + | 2.99E-01 |
| ^**^rs11228258 |  | 11 | 120667809 | 1.47E-06 | PPP6R3 | - | **2.37E-02** |
| ^**^rs11228292 |  | 11 | 120670838 | 5.24E-07 | PPP6R3 | - | **2.37E-02** |
| ^*^rs917727 |  | 7 | 120671689 | 2.26E-11 | FAM3C | - | None |
| ^***^rs6117672 |  | 20 | 120672559 | 3.70E-02 | BMP2/FUSIP1P2 | + | 1.48E-01/None |
| ^**^rs10896348 |  | 11 | 120686577 | 2.22E-06 | PPP6R3 | + | **2.37E-02** |
| ^***^rs12205789 |  | 6 | 120689912 | 2.66E-02 | RUNX2/CLIC5 | - | 1.34E-01/**7.55E-06** |
| ^**^rs9533147 |  | 13 | 120695318 | 4.22E-02 | FABP3P2/TNFSF11 | - | None/**9.35E-04** |
| ^**^rs9533156 |  | 13 | 120738380 | 5.96E-03 | TNFSF11 | + | **9.35E-04** |
| ^***^rs1159530 |  | 20 | 120754026 | 2.21E-02 | BMP2/FUSIP1P2 | + | 1.48E-01/None |
| ^**^rs11624512 |  | 14 | 120762001 | 2.69E-02 | RIN3 | + | **6.10E-03** |
| ^**^rs9533154 |  | 13 | 120766325 | 1.56E-03 | FABP3P2/TNFSF11 | - | None/**9.35E-04** |
| ^***^rs1159531 |  | 20 | 120785513 | 6.02E-03 | BMP2/FUSIP1P2 | + | 1.48E-01/None |
| ^***^rs7124513 |  | 11 | 120805815 | 1.40E-02 | NUDT8 | + | None |
| ^***^rs435260 |  | 21 | 120812738 | 2.27E-02 | RPL34P3/LOC266693 | + | None/None |
| ^***^rs6054768 |  | 20 | 120820357 | 1.47E-02 | BMP2/FUSIP1P2 | + | 1.48E-01/None |
| ^***^rs6038724 |  | 20 | 166262283 | 1.95E-02 | BMP2/FUSIP1P2 | - | 1.48E-01/None |
| ^**^rs1895701 |  | 2 | 166285202 | 2.70E-02 | FAM130A2/GALNT3 | - | None/1.13E-01 |
| ^***^rs599083 |  | 11 | 166285735 | 7.37E-03 | LRP5 | + | 2.34E-01 |
| ^***^rs2272196 |  | 7 | 166286360 | 1.28E-02 | CPED1 | - | 2.99E-01 |
| ^***^rs2247156 |  | 10 | 166291387 | 4.73E-03 | LOC439954/PBEF2 | - | None/None |
| ^***^rs2268177 |  | 1 | 166291490 | 3.09E-03 | CDC42 | - | **3.38E-02** |
| ^**^rs777355 |  | 2 | 166311333 | 1.46E-03 | FAM130A2/GALNT3 | - | None/1.13E-01 |
| ^**^rs948315 |  | 11 | 166314046 | 1.65E-06 | PPP6R3 | + | **2.37E-02** |
| ^**^rs1524503 |  | 7 | 166315239 | 4.90E-03 | CPED1 | + | 2.99E-01 |
| ^**^rs4988291 |  | 11 | 166315992 | 7.72E-07 | PPP6R3 | - | **2.37E-02** |
| ^*^rs6591341 |  | 11 | 166322823 | 1.87E-06 | PPP6R3 | - | **2.37E-02** |
| ^**^rs7786203 |  | 7 | 166326508 | 1.49E-02 | CPED1 | - | 2.99E-01 |
| ^**^rs4316515 |  | 11 | 166328882 | 7.48E-07 | PPP6R3 | + | **2.37E-02** |
| ^**^rs9661787 |  | 1 | 168557304 | 4.10E-03 | FMN2 | + | 1.13E-01 |
| ^*^rs12741884 |  | 1 | 178430631 | 6.60E-04 | WNT4/LOC343384 | + | 1.53E-01/None |
| ^***^rs2242747 |  | 21 | 185897656 | 4.48E-02 | RPL34P3/LOC266693 | - | None/None |
| ^***^rs875625 |  | 13 | 238640833 | 4.42E-02 | TNFSF11 | + | **9.35E-04** |
| ^*^rs6726821 |  | 2 | 238653318 | 5.39E-04 | FAM130A2/GALNT3 | + | None/1.13E-01 |
| ^***^rs9472536 |  | 6 | 238659259 | 1.84E-03 | RUNX2/CLIC5 | - | 1.34E-01/**7.55E-06** |

Column definition: SNP – single nucleotide polymorphisms; SNPs with ^*^ were reported by previous GWASs to be associated with lean mass; SNPs with ^**^ reside in the same LD block as previously reported SNPs associated with lean mass; SNPs with ^***^ were novel potential ALM-associated SNPs identified by cFDR; Chr – chromosome; Position – chromosome position; ccFDR – conjunction conditional false discovery rate; + – positive effect; - – negative effect; ALM – appendicular lean mass; BMD – bone mineral density; p.ALM – p values of gene expression validation analysis on human vastus lateralis muscle biopsies; None – not detected. Bold p values are those that are nominally significant (p < 0.05).
