# Supplementary material for: Enhanced Identification of Novel Potential Variants for Appendicular Lean Mass by Leveraging Pleiotropy With Bone Mineral Density
Source: Front Immunol. 2021 Apr 6;12:643894. doi: 10.3389/fimmu.2021.643894 (PMC8056257; doi:10.3389/fimmu.2021.643894)
Supplement: Supplementary file 6 [file Table_4.docx]

**Supplementary Table 4. Potential lean mass associated SNPs identified by previous GWASs ( https://www.ebi.ac.uk/gwas, Jan 2020).**

| PubmedID | Study | SNPs | Mapped Gene | Mapped Trait |
| --- | --- | --- | --- | --- |
| 23251661 | Novel genetic loci identified for the pathophysiology of childhood obesity in the Hispanic population. | rs589756 | MOXD1 | Lean body mass |
| 23251661 | Novel genetic loci identified for the pathophysiology of childhood obesity in the Hispanic population. | rs1056513 | INADL | Lean body mass |
| 19268274 | Genome-wide association and replication studies identified TRHR as an important gene for lean body mass. | rs7832552 | TRHR | Lean body mass |
| 23108985 | Suggestion of GLYAT gene underlying variation of bone size and body lean mass as revealed by a bivariate genome-wide association study. | rs2507838 | ZFP91-CNTF/GLYAT | Lean body mass |
| 28743860 | Bivariate genome-wide association meta-analysis of pediatric musculoskeletal traits reveals pleiotropic effects at the SREBF1/TOM1L2 locus. | rs6591341 | PPP6R3 | Bone density, Lean body mass |
| 28743860 | Bivariate genome-wide association meta-analysis of pediatric musculoskeletal traits reveals pleiotropic effects at the SREBF1/TOM1L2 locus. | rs3765350 | WNT4 | Bone density, Lean body mass |
| 28743860 | Bivariate genome-wide association meta-analysis of pediatric musculoskeletal traits reveals pleiotropic effects at the SREBF1/TOM1L2 locus. | rs12741884 | MIR4418/LOC101060363 | Bone density, Lean body mass |
| 28743860 | Bivariate genome-wide association meta-analysis of pediatric musculoskeletal traits reveals pleiotropic effects at the SREBF1/TOM1L2 locus. | rs6684375 | LOC101060363/LOC105376856 | Bone density, Lean body mass |
| 28743860 | Bivariate genome-wide association meta-analysis of pediatric musculoskeletal traits reveals pleiotropic effects at the SREBF1/TOM1L2 locus. | rs6726821 | CSRNP3/GALNT3 | Bone density, Lean body mass |
| 28743860 | Bivariate genome-wide association meta-analysis of pediatric musculoskeletal traits reveals pleiotropic effects at the SREBF1/TOM1L2 locus. | rs7672749 | MEPE/HSP90AB3P | Bone density, Lean body mass |
| 28743860 | Bivariate genome-wide association meta-analysis of pediatric musculoskeletal traits reveals pleiotropic effects at the SREBF1/TOM1L2 locus. | rs13245690 | CPED1 | Bone density, Lean body mass |
| 28743860 | Bivariate genome-wide association meta-analysis of pediatric musculoskeletal traits reveals pleiotropic effects at the SREBF1/TOM1L2 locus. | rs917727 | FAM3C | Bone density, Lean body mass |
| 28743860 | Bivariate genome-wide association meta-analysis of pediatric musculoskeletal traits reveals pleiotropic effects at the SREBF1/TOM1L2 locus. | rs12284933 | PPP6R3 | Bone density, Lean body mass |
| 28743860 | Bivariate genome-wide association meta-analysis of pediatric musculoskeletal traits reveals pleiotropic effects at the SREBF1/TOM1L2 locus. | rs9525638 | LOC105370177/TNFSF11 | Bone density, Lean body mass |
| 28743860 | Bivariate genome-wide association meta-analysis of pediatric musculoskeletal traits reveals pleiotropic effects at the SREBF1/TOM1L2 locus. | rs754388 | RIN3 | Bone density, Lean body mass |
| 28743860 | Bivariate genome-wide association meta-analysis of pediatric musculoskeletal traits reveals pleiotropic effects at the SREBF1/TOM1L2 locus. | rs7501812 | TOM1L2 | Bone density, Lean body mass |
| 28743860 | Bivariate genome-wide association meta-analysis of pediatric musculoskeletal traits reveals pleiotropic effects at the SREBF1/TOM1L2 locus. | rs2955382 | GID4 | Bone density, Lean body mass |
| 28724990 | Large meta-analysis of genome-wide association studies identifies five loci for lean body mass. | rs2943656 | LOC646736/LOC105373915 | Lean body mass |
| 28724990 | Large meta-analysis of genome-wide association studies identifies five loci for lean body mass. | rs2287926 | VCAN | Lean body mass |
| 28724990 | Large meta-analysis of genome-wide association studies identifies five loci for lean body mass. | rs4842924 | ADAMTSL3 | Lean body mass |
| 28724990 | Large meta-analysis of genome-wide association studies identifies five loci for lean body mass. | rs9936385 | FTO | Lean body mass |
| 28724990 | Large meta-analysis of genome-wide association studies identifies five loci for lean body mass. | rs9641123 | CALCR | Lean body mass |
| 28724990 | Large meta-analysis of genome-wide association studies identifies five loci for lean body mass. | rs1028883 | LOC105377816/LINC00393 | Lean body mass |
| 28724990 | Large meta-analysis of genome-wide association studies identifies five loci for lean body mass. | rs9991501 | HSD17B11 | Lean body mass |
