# Supplementary material for: Enhanced Identification of Novel Potential Variants for Appendicular Lean Mass by Leveraging Pleiotropy With Bone Mineral Density
Source: Front Immunol. 2021 Apr 6;12:643894. doi: 10.3389/fimmu.2021.643894 (PMC8056257; doi:10.3389/fimmu.2021.643894)
Supplement: Supplementary file 7 [file Table_5.docx]

**Supplementary table 5. GO Terms of genes corresponding to novel potential pleiotropic SNPs (adjP < 0.05)**

| GO term | GO ID | adjP | Genes |
| --- | --- | --- | --- |
| bone development | 60348 | 1.60E-03 | BMP2, LRP5, RUNX2 |
| regulation of glucocorticoid metabolic process | 31943 | 1.60E-03 | BMP2, GAL |
| osteoblast development | 2076 | 2.40E-03 | LRP5, RUNX2 |
| regulation of odontogenesis | 42481 | 2.40E-03 | BMP2, RUNX2 |
| regulation of hormone metabolic process | 32350 | 2.40E-03 | BMP2, GAL |
| positive regulation of fat cell differentiation | 45600 | 2.40E-03 | BMP2, LRP5 |

Column definition: GO – gene ontology term; adjP – p value adjusted by the multiple test adjustment.
